# Impact of Covid-19 Pandemic on the Effectiveness of Outpatient Counseling in Childhood Obesity Management

**DOI:** 10.3389/fendo.2022.879440

**Published:** 2022-07-04

**Authors:** Domenico Corica, Alessandra Li Pomi, Selenia Curatola, Giorgia Pepe, Annalisa Giandalia, Angelo Tropeano, Angela Alibrandi, Tommaso Aversa, Malgorzata Wasniewska

**Affiliations:** ^1^ Department of Human Pathology of Adulthood and Childhood “G. Barresi”, University of Messina, Messina, Italy; ^2^ Department of Clinical and Experimental Medicine , University of Messina, Messina, Italy; ^3^ Department of Economics, University of Messina, Messina, Italy

**Keywords:** childhood obesity, Covid-19, physical activity, lifestyle, nutrition

## Abstract

The Covid-19 pandemic drastically modified social life and lifestyle, in particular, among children and adolescents, promoting sedentary behaviors and unhealthy eating habits. The aims of this study were to assess the rate and the factors associated with outpatient drop-out in childhood obesity management, and to evaluate how the Covid-19 pandemic influenced weight status and lifestyle of children and adolescents with obesity. One hundred and forty-five children and adolescents with obesity were identified, including 80 subjects evaluated before the Covid-19 pandemic (group A) and 65 subjects in the period straddling the Covid-19 pandemic (group B). Anamnestic (family history of obesity, dietary habits, physical activity, screen time), socio-cultural (economic status, employment and schooling of parents, household composition, place of living) and clinical (weight, height, BMI, waist circumference) data were retrospectively analyzed for each subject in both groups at baseline (V0) and 12-months (V1) at in-person assessment. Glycemic and lipid profiles were assessed at V0. Drop-out rate did not differ significantly between the two groups. BMI SDS at V0 (OR=2.52; p=0.004), female sex (OR=0.41; p=0.035), and the presence of a single parent in the household (OR=5.74; p=0.033) significantly influenced drop-out in both groups. Weight loss between V0 and V1 was significantly greater among group A patients compared to group B (p=0.031). In group B, hours spent in physical activity significantly decreased from V0 to V1, being significantly lower than group A at V1; on the contrary, screen time significantly increased in the same period. The consumption of sugary drinks and snacks was significantly greater in group B than group A at V1. Our study documented that the Covid-19 pandemic, although not affecting the drop-out rate of obese children in a follow-up program, negatively influenced lifestyle and reduced the effectiveness of outpatient counseling in childhood obesity treatment.

## Introduction

The coronavirus disease (Covid-19), caused by Sars-CoV-2, firstly reported in late December 2019 in Wuhan, China, quickly became an emerging, rapidly evolving situation, spreading inevitably outside China and the Asian continent, and was declared a pandemic in March 2020. Governments of several countries worldwide enforced emergency measures and a lockdown policy, including closure of schools and many other activities, to slow infection spread down. In Italy, the Government imposed a lockdown from March 9, 2020 to May 4, 2020, which was followed by a gradual restart of activities, although school activities continued with distance learning through to the end of the school year, and most extracurricular activities previously attended by children did not restart after closing. These safety measures radically modified social life and drastically changed people’s lifestyle, in particular for children and adolescents (1). Youth lifestyle behaviors were significantly affected due to extended school closures and home confinement, leading to a reduction in physical activity in favor of a sedentary lifestyle, particularly impacting children and adolescents with obesity. A sedentary lifestyle, characterized by increased time spent watching TV or playing video games, promotes unhealthy behaviors which favor the onset or worsening of obesity, such as high consumption of fast food and sugar-sweetened beverages, as well as sleep disorders (2, 3). These issues can be further exacerbated by stress due to isolation, which can result in increased food intake, with a particular focus on highly palatable foods (sugary, salty and high-calorie foods), an increase in emotional instability and in overall worsening of the quality of life (2). On the other hand, it has been suggested that lockdown and social distancing policies may have resulted in a reduction in the risk of developing or worsening obesity as a result of reduced out-of-home meal occasions (restaurants and fast food) (4, 5). Therefore, the effects of the Covid-19 pandemic on pediatric weight status are not completely clear. Moreover, many studies have based their results on questionnaires or telephone interviews rather than in-person assessments. In this scenario, the effectiveness of the outpatient approach for pediatric obesity, already burdened by a high drop-out rate, may decrease due to restrictions imposed by the Covid-19 pandemic.

The aims of this study were to assess the rate and the factors associated with outpatient drop-out by comparing two groups of children and adolescents with obesity evaluated before and during the Covid-19 pandemic, and to evaluate how the Covid-19 pandemic influenced the weight status and lifestyle of these subjects.

## Materials and Methods

### Study Population

This is a single-center, observational, retrospective study. Patient inclusion criteria were age ranged between 5 and 16 years, and BMI ≥ +2 standard deviation score (SDS) at baseline, in accordance with the definition of obesity by the World Health Organization (WHO) for children from the age of 5 years. Exclusion criteria were genetic and/or endocrine causes of obesity, chronic diseases, and chronic pharmacological therapies.

Children and adolescents, referred to our pediatric endocrinology outpatient clinic for obesity, who completed annual follow-ups, were divided into two groups: group A included subjects evaluated before the Covid-19 pandemic and group B included subjects followed in the period straddling the Covid-19 pandemic. Specifically, each subject belonging to the two groups was evaluated retrospectively at a first in-person evaluation (V0) and at a 12-month follow-up in-person visit (V1). Group A patients underwent V0 assessment between October and November 2018 and V1 evaluation between October and November 2019, while patients of group B underwent V0 between January and February 2020 and V1 evaluation between January and February 2021.

### Anamnestic Assessment

Anamnestic information was obtained by evaluating both face-to-face interview with parents, conducted during appointments, and medical records. In particular, information regarding the following was collected: family history for obesity in parents, socio-cultural aspects including household composition (complete or incomplete with separated parents), employment of both parents (yes or no), family economic status (low/moderate or high status) estimated indirectly in relation to the employment of one or both parents, level of education of both parents (years of school attendance including university), place of living (town or village). In addition, for each patient assessed, at both V0 and V1, information regarding the following was collected: hours spent daily on video games, TV, tablets, smartphone (screen time), the practice of physical activity (yes or no) and the number of hours per week spent on physical activity, hours of sleep during the school period and during the weekend, number of daily meals, sugar-sweetened beverage intake frequency (every day, once a week, many times a week, very rarely, never), snack intake frequency (every day, once a week, many times a week, very rarely, never), improvement in eating habits (yes or no) between V0 and V1 defined in relation to change in consumption of snacks and sugary drinks reported by parents during the face-to-face interview at V1.

### Clinical and Biochemical Evaluation

Clinical information was obtained from medical records. At V0 and V1, a physical evaluation was performed according to standardized procedures, including assessment of height, weight, BMI, BMI SDS, waist circumference (WC), WC-to-height ratio (WHtR), systolic and diastolic blood pressure (6). Weight loss was defined as a reduction in BMI SDS > 0.25 (7).

At V0, children of both groups underwent fasting biochemical assessments (oral glucose tolerance test (OGTT), lipid profile, thyroid, kidney and liver function tests, c-reactive protein), according to methodology previously described (8). Homeostasis model assessment of insulin resistance (HOMA-IR) was calculated as fasting insulin (mIU/L) × fasting glucose (mg/dL)/405 (9).

### Statistical Analysis

Numerical data were expressed as mean and standard deviations score (SDS) and the categorical variables as absolute frequencies and percentages. The parametric approach was used since most numerical variables were not normally distributed, as verified by the Kolmogorov Smirnov test.

To compare group A and group B features, the Mann Whitney test was applied with reference to numerical parameters, and the Chi Square test with reference to categorical variables both at V0 and V1.

To perform intra-group comparison analysis between two time-points (V0 and V1), both in group A and group B separately, we applied the Wilcoxon test for numerical parameters and Mc Nemar’s test for dichotomous variables. Multivariate logistic stepwise regression models were estimated to identify significant predictors of drop-out (model covariates: belonging to group A or B, obesity of mother and father, economic status, education level of parents, family structure, parents’ employment, place of living, sex, and age, pubertal stage, BMI SDS at V0) and predictors of weight loss (model covariates: belonging to group A or B, obesity of mother and father, economic status, education level of parents, family structure, parents’ employment, place of living, sex, age and pubertal stage at V0, and hours of sleep, sugar-sweetened beverage and snack intake frequency, screen time and practice and weekly hours of physical activity at V1). The results were expressed as Odds Ratio (OR), 95% confidence interval and p-value. Statistical analysis was performed using IBM SPSS Statistics for Windows, Version 22 (Armonk, NY, IBM Corp.). A *p*-value < 0.05 was considered statistically significant.

## Results

One hundred and forty-five children and adolescents with obesity were evaluated, including 80 subjects in group A and 65 subjects in group B. Subjects of the two groups were matched for age, sex, pubertal stage, BMI and BMI SDS (**Table 1**). Thyroid, liver and kidney function tests were normal in the entire population. At V0, there were no significant differences between the two groups with regard to clinical and biochemical parameters (**Table 1**). Socio-cultural and lifestyle characteristics of the two groups at V0 and V1 are shown in **Table 2**. Subjects who continued outpatient follow-up, reevaluated at V1, were 43.8% for group A and 35.4% for group B. The drop-out rate did not differ significantly between the two groups (56.2% vs 64.6%; p=0.410). BMI SDS at V0, female sex, and the presence of a single parent in the household (incomplete) significantly influenced drop-out in both groups (**Table 3**).

**Table 1:** Comparison analysis between group A and group B on baseline clinical and biochemical parameters.

	Group A (n=80)	Group B (n=65)	p
Age (year)	11.6 ± 2.3	11.8 ± 2.7	0.738
Sex (male/female)	36/44	29/36	0.963
Pubertal/pre-pubertal	55/25	43/22	0.740
Height SDS	0.36 +/- 1.066	0.38 +/- 1.14	0.913
BMI (kg/m^2^)	29.75 +/- 4.37	29.79 +/- 4.76	0.962
BMI SDS	3.00 +/- 0.57	3.12 +/- 1.07	0.455
WC (cm)	87.56 +/- 9.41	89.78 +/- 12.01	0.271
WHtR	0.89 +/- 0.07	0.89 +/- 0.08	0.924
SBP (mmHg)	114.80 +/- 11.84	116.26 +/- 12.43	0.510
DBP (mmHg)	71.31 +/- 10.66	71.16 +/- 10.94	0.940
Fasting glucose (mg/dl)	94.29 +/- 7.31	94.69 +/- 10.87	0.798
Fasting insulin (mIU/L)	23.49 +/- 16.78	20.85 +/- 10.60	0.250
HbA1c	5.44 +/- 0.38	5.41 +/- 0.41	0.061
2h-glucose (mg/dl)	123.76 +/- 20.64	122.32 +/- 29.46	0.742
2h-insulin (mIU/L)	127.07 +/- 100.22	104.68 +/- 60.95	0.100
HOMA-IR	5.52 +/- 4.02	4.91 +/- 2.63	0.407
Total cholesterol (mg/dl)	168.11 +/- 26.54	174.51 +/- 29.52	0.177
HDL cholesterol (mg/dl)	47.77 +/- 10.17	51.14 +/- 12.49	0.083
LDL cholesterol (mg/dl)	96.36 +/- 22.71	100.12 +/- 29.77	0.347
Triglycerides (mg/dl)	97.09 +/- 48.81	90.52 +/- 37.73	0.363
TSH (μUI/ml)	3.08 +/- 1.35	2.68 +/- 1.42	0.087
FT4 (ng/dl)	1.26 +/- 0.15	1.22 +/- 0.14	0.144
ALT (U/L)	24.59 +/- 28.58	23.34 +/- 15.70	0.739
AST (U/L)	26.56 +/- 31.15	21.00 +/- 6.89	0.124
GGT (U/L)	15.16 +/- 7.02	14.75 +/- 8.23	0.795
Uric acid (mg/dl)	4.92 +/- 1.25	5.05 +/- 1.16	0.516
CRP (mg/dl)	3.25 +/- 7.54	2.89 +/- 3.16	0.701

Subjects evaluated before the Covid-19 pandemic (Group A); subjects evaluated in the period straddling the Covid-19 pandemic (Group B), Body mass index (BMI), standard deviation score (SDS), waist circumference (WC), WC-to-height ratio (WHtR), systolic blood pressure (SBP), diastolic blood pressure (DBP), alanine aminotransferase (ALT), aspartate aminotransferase (AST), Gamma-Glutamyl Transferase (GGT), glycated haemoglobin (HbA1c), 120-minutes OGTT glucose levels (2h- glucose), 120-minutes OGTT insulin levels (2h-insulin), model assessment of insulin resistance (HOMA-IR), free thyroxine (FT4), thyroid stimulating hormone (TSH), c-reactive protein (CRP).

**Table 2:** Comparison analysis between groups on baseline (2A) and 12-month (2B) anamnestic assessment.

2A			
	Group A (n=80)	Group B (n=65)	p-value
Physical activity (yes/no)	50/30	40/25	0.608
Physical activity (hours)	2.22 +/- 2.35	1.69 +/- 1.36	0.091
Screen time (hours)	3.81 +/- 2.23	3.25 +/- 1.58	0.098
Number of meals	5.05 +/- 0.70	4.68 +/- 0.86	0.007
Sleep school (hours)	8.33 +/- 1.41	7.74 +/- 0.94	0.005
Sleep weekend (hours)	9.35 +/- 1.42	8.60 +/- 1.09	0.001
Age of obesity onset (years)	5.67 +/- 2.65	6.27 +/- 2.88	0.214
Obesity of mother (yes/no)	42/38	35/30	0.774
Obesity of father (yes/no)	39/41	27/38	0.096
Family economic status (Low/moderate-high)	32/48	26/39	0.738
Household composition (complete/incomplete)	63/17	62/3	0.004
Employment of mother (yes/no)	35/45	25/40	0.410
Employment of father (yes/no)	63/17	50/15	0.291
Place of living (town/village)	39/41	29/36	0.620
Mother’s education (school years)	11.22 +/- 3.37	9.90 +/-2.96	0.019
Father’s education (school years)	11.22 +/- 3.64	10.21+/- 3.10	0.091
**2B**			
	**Group A (n=35)**	**Group B (n=24)**	**p-value**
Physical activity (yes/no)	20/15	2/22	0.001
Physical activity (hours)	1.82 +/- 1.54	0.46 +/- 1.06	0.011
Screen time (hours)	2.76 +/- 1.53	5.45 +/- 2.11	0.000
Number of meals	4.97 +/- 0.17	4.81 +/- 1.12	0.519
Sleep school (hours)	8.45 +/- 0.95	7.50 +/- 1.05	0.002
Sleep weekend (hours)	9.40 +/- 1.31	8.35 +/- 1.18	0.004

Subjects evaluated before the Covid-19 pandemic (Group A); subjects followed in the period straddling the Covid-19 pandemic (Group B).

**Table 3:** Stepwise logistic regression analysis for drop-out and weight loss outcomes.

Predictors	OR	95%CI	*p*
**DROP-OUT**			
Sex (Female)	0.41	0.18 - 0.94	0.035
Household composition (incomplete)	5.74	1.15 - 28.58	0.033
BMI SDS (V0)	2.52	1.35 - 4.70	0.004
**WEIGHT LOSS**			
Group (A)	0.16	0.32 - 0.82	0.028
Weekly hours of physical activity (V1)	2.27	1.05 – 4.96	0.038

Odds ratio (OR), 95% confidence interval (95%CI), baseline assessment (V0), 12-month assessment (V1) subjects evaluated before the Covid-19 pandemic (Group A).

Intra-group comparison analysis between the V0 and V1 evaluations documented a significant decrease in BMI SDS in group A (3.0 ± 0.57 vs 2.59 ± 0.7; p=0.008), while among group B patients BMI SDS did not change significantly (3.12 ± 1.07 vs 2.97 ± 1.19; p=0.454) and BMI in absolute value increased significantly (29.79 ± 4.76 vs 30.98 ± 4.82; p=0.003). Moreover, comparison analysis between the groups documented a significantly higher BMI SDS among group B patients compared with those belonging to group A at V1 (30.98 ± 4.82 vs 28.51 ± 4.13; p=0.041). Consistently, weight loss was significantly greater among group A patients compared to group B at V1 (67.6% vs 32.4%; p=0.031).

Hours spent in physical activity decreased significantly in group B from V0 to V1 (1.69 ± 1.36 vs 0.46 ± 1.06; p= 0.001), whereas it did not change significantly between the two assessments in group A (2.22 ± 2.35 vs 1.82 ± 1.54; p= 0.068). Moreover, hours spent in physical activity, which were not significantly different between the two groups at V0 (**Table 2A**), were significantly greater among group A subjects compared to subjects of group B at V1 (**Table 2B**). In addition, the number of subjects who had engaged in regular physical activity between V0 and V1 was significantly higher in group A than group B (**Table 2B**).

Screen time decreased significantly in group A from V0 to V1 (3.81 ± 2.23 hours vs 2.76 ± 1.53; p=0.000), whereas it increased significantly in group B between the two assessments (3.25 ± 1.58 vs 5.45 ± 2.11 hours; p=0.002). Moreover, screen time, which was not significantly different between the two groups at V0 (**Table 2A**), was significantly greater among patients in group B at V1 evaluation (**Table 2B**).

Improvement in eating habits between V0 and V1 was greater in group A than in group B (86% vs 35%, p=0.000). Consistently, the intragroup comparison analysis showed a significant reduction in snack and sugary drink intake between V0 and V1 in group A, whereas no significant difference was documented in group B (**Table 4**). Moreover, comparison analysis between the groups documented a significantly more frequent consumption of sugary drinks and snacks in group B compared to group A at V1 (**Table 5**).

**Table 4:** Intragroup comparison analysis concerning snack (4A) and sugar-sweetened beverage intake (4B) between baseline (V0) and 12-month evaluation (V1).

4A						
	Group A			Group B		
INTAKE FREQUENCY	V0 (N=80)	V1 (N=35)	p	V0 (N=65)	V1 (N=23)	p
**Never**	3.8%	25.7%	0.000	9.3%	21.7%	0.125
**Every day**	27.5%	8.6%	0.024	44.6%	26.1%	0.121
**Once a week**	28.8%	2.9%	0.002	13.8%	17.4%	0.677
**Many times a week**	21.2%	11.4%	0.212	18.5%	30.4%	0.236
**Very rarely**	18.8%	51.4%	0.000	13.8%	4.4%	0.224
**4B**						
	**Group A**			**Group B**		
**INTAKE FREQUENCY**	**V0 (N=80)**	**V1 (N=35)**	**p**	**V0 (N=65)**	**V1 (N=23)**	**p**
**Never**	7.5%	34.3%	0.000	15.4%	17.4%	0.822
**Every day**	25%	5.7%	0.016	27.7%	26.8%	0.934
**Once a week**	22.5%	2.9%	0.009	18.4%	4.3%	0.102
**Many times a week**	11.2%	8.6%	0.675	18.4%	34.3%	0.119
**Very rarely**	33.8%	48.6%	0.135	20%	17.4%	0.787

Chi-square test was applied.

Subjects evaluated before the Covid-19 pandemic (Group A); subjects followed in the period straddling the Covid-19 pandemic (Group B).

**Table 5:** Between-group comparison analysis regarding sugar-sweetened beverage (5A) and snack (5B) intake between baseline (V0) and 12-month evaluation (V1).

5A						
	V0			V1		
INTAKE FREQUENCY	Group A (N=80)	Group B (N=65)	p	Group A (N=35)	Group B (n=23)	p
**Never**	7.5%	15.4%	0.132	34.3%	17.3%	0.160
**Every day**	25%	27.7%	0.714	5.7%	26.8%	0.025
**Once a week**	22.5%	18.4%	0.545	2.9%	4.3%	0.771
**Many times a week**	11.2%	18.4%	0.221	8.6%	34.3%	0.015
**Very rarely**	33.8%	20.0%	0.065	48.6%	17.4%	0.017
**5B**						
	**V0**			**V1**		
**INTAKE FREQUENCY**	**Group A (N=80)**	**Group B (N=65)**	**p**	**Group A (N=35)**	**Group B (n=23)**	**p**
**Never**	3.8%	9.3%	0.176	25.7%	21.7%	0.729
**Every day**	27.5%	44.6%	0.033	8.6%	26.1%	0.074
**Once a week**	28.8%	13.8%	0.031	2.9%	17.4%	0.057
**Many times a week**	21.2%	18.5%	0.687	11.4%	30.4%	0.073
**Very rarely**	18.8%	13.8%	0.422	51.4%	4.3%	0.000

Chi-square test was applied.

Subjects evaluated before the Covid-19 pandemic (Group A); subjects followed in the period straddling the Covid-19 pandemic (Group B).

Hours spent in physical activity (OR=2.27, p=0.038) and belonging to group A (OR=0.16, p=0.028) were the only factors influencing weight loss (**Table 3**).

## Discussion

The Covid-19 pandemic is causing significant health, social, and economic implications that have drastically modified social life. These include the interruption of regular school attendance and extracurricular activities for children and adolescents, which has led to an increase in sedentary behaviors and unhealthy eating habits, especially during the lockdown period. The pandemic has promoted a sedentary lifestyle and high consumption of fast food and sugar-sweetened beverages (10). Moreover, Xiang et al. documented a drastic decrease in median time spent in physical activity, showing a 435 min/week reduction on average, and a significant increase in screen time during the Covid-19 pandemic (11). Unhealthy diet, excessive screen time, insufficient time spent on physical activity and nightly rest are known behavioral risk factors for the onset or worsening of obesity (12).

Our study documented that the Covid-19 pandemic negatively influenced the lifestyle of children and adolescents with obesity in outpatient follow-up, promoting a worsening severity of overweight. In our study, although there was no increased drop-out rate during the pandemic period, a significant reduction in time spent on physical activity and a significant increase in screen time and junk food consumption was documented after one year of follow-up among patients followed during the pandemic period compared to those in follow-up before the Covid-19 pandemic.

With a telephone interview conducted three weeks after the beginning of lockdown in Italy, Pietrobelli et al. assessed the implications of “staying home” on food intake and daily habits in children followed at their clinics, to document whether indeed young people with obesity show unfavorable trends in lifestyle behaviors when away from structured school activities and confined to their homes. Specifically, the authors documented a significant increase in the number of meals consumed per day and an increased intake of crisps, red meat, and sugary drinks as reported by parents during the telephone interview. In addition, sleep and screen time had increased significantly, while sports time had decreased significantly (1).

Similarly, in another study based on data from a questionnaire completed online by parents, Pujia et al. reported a significant increase in consumption of “comfort food”, including packaged sweet snacks, chocolate, ice cream, dessert, but also processed meat, bread, pizza, and bakery products, in 439 Italian children and adolescents. Among these subjects, approximately 60% reported an increase in body weight, which was significantly greater in adolescents than in children (13). Consistently, Appelhans et al. demonstrated a significant weight gain among low-income, racial minority, obese children followed during the Covid-19 period compared to those followed in the pre-pandemic period at 12-month assessment (14).

These findings support the thesis that the Covid-19 pandemic exacerbated the risk factors for the so-called “summer break-associated weight gain” due to school closures, which, in this case, was imposed during the lockdown (15). Previous studies highlighted that weight control programs are less effective among youth while they are at home than when they are engaged at school. A study by von Hippel et al. analyzed the effects of school versus non-school environments on overweight in childhood, demonstrating that BMI gain was faster during summer vacations compared to the in-session school year (16). This finding may be due to the opportunity provided by school environments for structure and routines for mealtimes, physical activity, and sleep schedule, the three predominant factors that, if altered, may increase the risk of obesity (17).

Worldwide, a significant reduction in daily physical activity has been demonstrated since the pandemic onset (18). Moreover, besides contributing to weight gain, the drastic reduction in physical activity during the pandemic and the concomitant overuse of electronic devices contributed to a significant increase in diagnoses of sleep disorders and psychiatric disorders, such as anxiety and depression, among children and adolescents (19, 20).

We documented that physical activity was the main factor influencing weight status and that the Covid-19 pandemic resulted in a significant reduction in time spent on physical activity among children with obesity. Performing physical activity, even at home, plays a crucial role in containing the increase of childhood obesity. Children and adolescents should perform 1 hour of daily physical activity of moderate to vigorous intensity to promote and maintain good health and weight in the normal range; in addition, activities to strengthen the musculoskeletal system should be included at least 3 times per week. In our study, patients followed at the outpatient clinic in the pandemic period had performed significantly fewer hours of physical activity than patients followed in the pre-pandemic period at the annual follow-up. In this pandemic period, physical activity at home has to be promoted, using the same electronic devices that are often the cause of sedentariness, with online physical activity classes, exercise apps on mobile devices, or video games that have a physical activity component.

With regard to the effects of the Covid-19 pandemic on the social life of children and adolescents, it should be considered that, besides the period of strict lockdown, the resumption of school and extracurricular activities has been gradual, and to date in many cases have not returned to pre-pandemic levels. Due to the persistence of the Covid-19 pandemic and related restrictions, school closures and even restrictions on social activities for children and adolescents have frequently reoccurred over the last two years, promoting an unhealthy lifestyle. Moreover, a regular outpatient follow-up of children and adolescents with obesity, which often requires closely-timed visits to be effective, has been made difficult by the above-mentioned restrictions. Elbarbary et al., in an International Cross-Sectional Electronic Survey distributed to the global network of endocrine societies, estimated a 41.5% delay in obesity diagnosis and a perceived worsening in obesity management in 83% of cases due to the COVID-19 pandemic (21). Our study showed that, although outpatient follow-up would not appear to have been compromised, its efficacy was lower in obese patients followed during the pandemic period compared with those followed in the pre-pandemic period, due to the increase of an unhealthy lifestyle characterized by a significant reduction in physical activity and increased time spent watching TV, playing video games and using electronic devices, and by an increased consumption of junk food. Continuity of care is a key aspect in the treatment of obesity, so discontinuation or limitation of outpatient follow-up and of activities that promote movement due to pandemic restrictions has negatively affected care in childhood obesity. In this context, telemedicine, if properly regulated, could be an effective solution to continue the follow-up of obese patients, relying on a chronic model of care. This option will need to be considered if restrictions are to continue, having the potential to play a key role in long-term multidisciplinary management of childhood obesity (22, 23). The results of our study may not fully reflect the weight status of individuals who are not similarly followed by professional medical care providers, since only patients who attended our pediatric endocrinology clinic before and during the pandemic were included in the study. Therefore, the study results should be considered in light of this possible limitation. The major strengths of this study consist in the study design, carried out by comparing two homogeneous groups assessed before and during the pandemic, and in the sourcing of data from in-person evaluation.

## Conclusions

Our study documented that the Covid-19 pandemic, although not affecting the drop-out rate of obese children, negatively influenced lifestyle, causing a decrease of time spent in physical activity and a significant worsening of eating habits, ultimately reducing the effectiveness of outpatient counseling. The persistent restriction of school and extracurricular activities due to the Covid-19 pandemic is likely to worsen obesity and to further reduce the effectiveness of the outpatient approach to treating childhood obesity. Continuity of care is crucial for successful treatment of childhood obesity. Strategies to manage unhealthy weight gain are urgently needed in the context of Covid-19. With this in mind, institutions and parents should recognize this growing problem by promoting physical activity and healthy eating at school and in extracurricular settings, by supporting telemedicine when necessary, and by encouraging anti-Sars-Cov-2 vaccination in children and adolescents.

## Data Availability Statement

The raw data supporting the conclusions of this article will be made available by the authors, without undue reservation.

## Ethics Statement

Ethical review and approval was not required for the study with human participants in accordance with local legislation and institutional requirements for retrospective studies, but notification was sent to the Local Ethics Committee regarding the retrospective evaluation of the data. Written informed consent from the participants' legal guardian was not required for retrospective study in accordance with national legislation and institutional requirements. The study was conducted according to the guidelines of the Declaration of Helsinki.

## Author Contributions

DC and MW contributed to conception and design of the study. AL, AT, AG, SC performed literature research. DC, TA, GP, SC, AL organized the database and prepared the tables. AA performed statistical analysis. DC, AL and MW wrote the first draft of the manuscript. DC and MW wrote the final version of the manuscript. All authors contributed to manuscript revision, read, and approved the submitted version.

## Conflict of Interest

The authors declare that the research was conducted in the absence of any commercial or financial relationships that could be construed as a potential conflict of interest.

## Publisher’s Note

All claims expressed in this article are solely those of the authors and do not necessarily represent those of their affiliated organizations, or those of the publisher, the editors and the reviewers. Any product that may be evaluated in this article, or claim that may be made by its manufacturer, is not guaranteed or endorsed by the publisher.
